# Effective Cryopreservation of Post Mortem-Collected Roe Deer Gametes by Evaluation of Post-Thaw Oocyte and Sperm Characteristics and In Vitro Fertilization

**DOI:** 10.3390/ani15162335

**Published:** 2025-08-09

**Authors:** Anna Justyna Korzekwa, Elena Buzan, Bostjan Pokorny, Gulsum Ummu Boztepe, Marek Lecewicz, Władysław Kordan

**Affiliations:** 1 Reasearch Team of Biodiversity Protection, Institute of Animal Reproduction and Food Research of Polish Academy of Sciences (IAR&FR PAS), Trylinskiego 18 Str., 10-683 Olsztyn, Poland; 2 Faculty of Mathematics, Natural Sciences and Information Technologies, University of Primorska, Glagoljaška 8, 6000 Koper, Slovenia; 3 Faculty of Environmental Protection, College of Environmental Protection, Trg Mladosti 7, 3320 Velenje, Slovenia; bostjan.pokorny@fvo.si; 4 Slovenian Forestry Institute, Večna Pot 2, 1000 Ljubljana, Slovenia; 5 University Centre of Veterinary Medicine JU-UA, University of Agriculture in Kraków, 31-120 Krakow, Poland; 6 Department of Animal Biochemistry and Biotechnology, Faculty of Animal Bioengineering, University of Warmia and Mazury in Olsztyn, Oczapowskiego 5, 10-957 Olsztyn, Poland; mlecew@uwm.edu.pl (M.L.); wladyslaw.kordan@uwm.edu.pl (W.K.)

**Keywords:** roe deer, semen, oocytes, fertilization

## Abstract

The aim was to evaluate the effectiveness of semen cryopreservation and oocyte vitrification in roe deer as a potential method of gamete preservation for endangered deer species. Sperm were isolated from the cauda epididymis of fourteen bucks (*n* = 14). The motility measure (CASA) and morphology of fresh semen (FS) and frozen–thawed semen (TS) were compared. A hyaluronic binding assay was used to distinguish between mature FS spermatozoa expressing hyaluronan receptors and immature FS lacking these receptors, and the mitochondrial membrane potential (MMP) in TS was determined (flow cytometry). A Sperm–Hyaluronan Binding Assay (HBA) showed a viability rate of 61.9% in FS and 78.2% in TS. Oocytes received from eight does (*n* = 8) underwent a viability test and vitrification, and fresh oocytes from the other eight does (*n* = 8) were fertilized with TS and embryos were cultured until the blastocyst stage. The number of isolated oocytes, cumulus–oocyte complexes (COCs), cleaved embryos, and expanded blastocysts was evaluated. Higher percentages of morphological factors (acrosome, head, midpiece, and tail shape) were observed in FS compared to TS, whereas the motility and progressive movement were greater in TS (*p* ≤ 0.001). The viability was 50.5% and MMP was 40.6% in TS. A total of 311 oocytes were collected and from 150 COCs and 125 blastocysts developed. The viability of thawed oocytes after vitrification was 81%. The viability of vitrified oocytes and cryopreserved sperm confirmed the effectiveness of freezing protocols and highlights the potential for their implementation in other deer species.

## Simple Summary

Endangered animals need protection, including the preservation of gametes. We evaluated the protocol of semen cryopreservation and oocyte vitrification in common roe deer for its future implementation in other species. The received viability rate was almost 62% in fresh semen but frozen–thawed semen viability was also high—50.5%. We performed in vitro fertilization and from 150 cumulus–oocyte complexes, 125 blastocysts developed. The viability of oocytes after vitrification was 81%. Morphology, motility, progressive movement, and viability comparisons between fresh and frozen–thawed semen, and the high viability of fresh and vitrified oocytes indicate that effective freezing protocols were established, which suggests the potential for implementation in other deer species.

## 1. Introduction

European roe deer (*Capreolus capreolus*) taxonomically belong to the subfamily Capreolinae [1]. Currently, deer are expanding throughout Europe as one of the most common and numerous wild ungulates belonging to the game species [2]. This species is found in a wide range of environmental and climatic conditions, and the intrinsic and simultaneously uncontrolled nature of these conditions causes variability among fitness traits. In fact, the different features of the landscape and available resources lead to differences in size and weight and consequently in their ecology and physiology [3]. Three main mtDNA lineages of European roe deer have been identified, named according to their geographical distribution: western, central, and eastern [4,5]. In 1809, Lamarck postulated that subsequent generations acquired environmentally induced characteristics from their ancestors in a phenotype [6]. In addition, the origin of anthropogenic and environmental changes may affect the genotype and physiological processes, including species reproduction [7,8]. Developing successful techniques for preserving gametes could greatly benefit the European roe deer and serve as a pioneering model for reproductive technologies for various other species, especially those under protection, such as Bawean deer (*Axis kuhlii*), giant muntjac (*Muntiacus vuquangensis*), water deer (*Hydropotes inermis*), and Patagonian huemul (*Hippocamelus bisulcus*), which have been identified in the International Union for Conservation of Nature’s Red List of Threatened Species. Assisted reproductive technology (ART) prevents the loss of genetic diversity in small populations, enabling equal contributions of all individuals to future generations [9]. In addition, the collection and cryopreservation of gametes could significantly reduce the necessity of capturing wild animals or moving animals in captivity by creating gamete banks [10]. Moreover, due to the annual hunting season for wild ruminants in Europe, it is not possible to collect the experimental material—the reproductive tract—from females and males at the same time; thus, evaluation of gamete preservation would be helpful for experiments aiming to develop in vitro fertilization (IVF).

Roe deer exhibit a well-defined seasonal pattern of reproductive activity, with a mating phase (rut) in July and August [11]. After mating, testicular regression occurs in adult males, which involves a significant reduction in their testicular mass by about 20% [12] and a decrease in body mass (7.5%) [13]. Moreover, roe deer exhibit a unique embryonic diapause—a period during which an embryo is suspended at the blastocyst stage, where the blastocyst either expands at a very slow rate or remains totally quiescent [14]. Although semen cryopreservation in other deer species (e.g., red deer) is currently practiced and is well described [11,15], in roe deer, this process has only been conducted twice. Furthermore, the parameters of roe deer semen have been characterized only when fresh and stored in a chilled state [16,17]. Spermatozoa cryoconservation in roe deer was performed by Prieto-Pablos et al. [16], who studied the effects of a chilling, freezing, and post-thaw incubation processes on the quality of semen collected with electroejaculation, and by Bóveda et al. [17], who tested two methods for the cryopreservation of epididymal semen collected at different times of the year. Animal species have many different traits related to their reproductive biology; for example, there is wide variation in the lipid and protein composition of the sperm membrane and its permeability to water and solutes. Such differences contribute to variances in the survival of spermatozoa through the chilling, freezing, and thawing processes [18]. Additionally, factors arising from the collection of the material itself, such as the hormonal status of the animal at the time of sperm collection or changes in the proteome resulting from different methods of sperm collection (electroejaculation and post mortem from epididymes), also change the sperm’s cryoresistance [19,20]. The specific features and process for successfully freezing roe deer oocytes using the rapid cooling method, known as vitrification, have not yet been determined. Vitrification results in a glass-like structure without ice crystals. During vitrification, oocytes are exposed to relatively high concentrations of permeating and non-permeating cryoprotectants, which act by lowering the freezing point of the cytoplasm and causing oocyte dehydration. Once equilibrated, the sample is cooled at ultra-rapid rates by plunging it directly into liquid nitrogen. The only exception considering the characteristics of roe deer oocytes was found when evaluating zona pellucida during oocyte maturation [21].

We hypothesized that the preservation of roe deer gametes could be an ART for deer species, including endangered deer, and thus the preservation protocol should be evaluated. This will be the basis for continuing research concerning reproduction, such as pregnancy development, e.g., determination of the unique embryonic diapause phenomenon from the embryo side. The results will be enriched in a future study with different roe deer ecotypes for indication as to how the environment shapes the reproductive potential of roe deer in different biotopes.

The aim of this study was to determine (i) the characteristics of fresh and cryopreserved spermatozoa and oocytes, (ii) the competence (maturation) of spermatozoa and oocytes for IVF, and (iii) the fertilization ability of spermatozoa and oocytes.

## 2. Materials and Methods

Testes and ovaries were collected post mortem from free-ranging adult males (*n* = 14) and females (*n* = 16) immediately (up to 30 min) after they were hunted. The age of animals (3 to 5 years) was estimated according to the tooth eruption stage and abrasion of teeth, assessed for each individual by an experienced hunter. The experimental material was collected during the hunting seasons in 2021 and 2023 (males—from 15 July to 20 August 2021 and 2022; females—from 5 November to 5 January 2022 and 2023) in central Poland—the Ciechanów Forest District (hunting license: ZG7521-3/2021/2022/2023)—and in northeast Poland—the area managed by the Hunting Association “Śniardwy” (hunting license: ZG 6721/2021/2022/2023).

**Figure 1:**
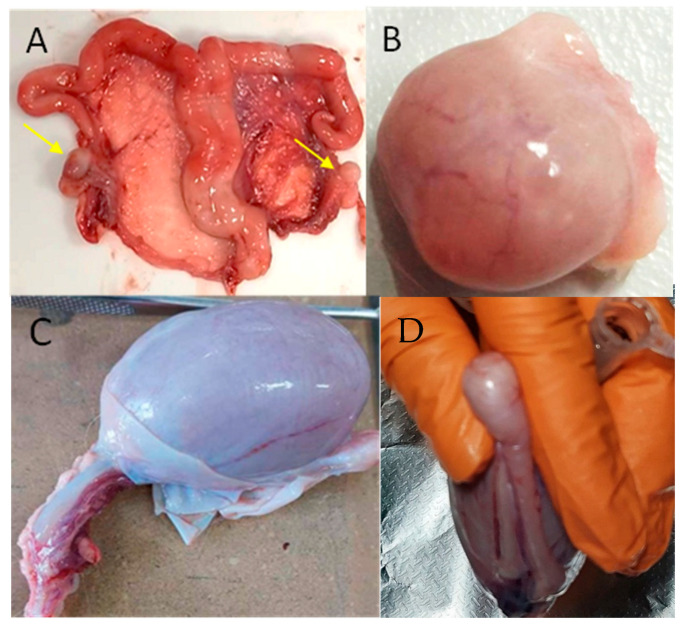
(**A**) Roe deer female reproductive tract—arrows indicate both ovaries. (**B**) Ovary. (**C**) Testis. (**D**) Epididymis.

No animals were culled for the purpose of this study, as they were harvested during regular hunting activities for management purposes prescribed by national regulations. The hunter collected the reproductive tracts from each animal immediately after they were shot. All does included in the study had ovulated, which was confirmed by the presence of at least one corpus luteum on the ovary. The scrotums, including the testicles and epididymides, were transported on ice, and the ovaries were transported in saline warmed to 18 °C to the laboratory for further procedures over max. 40 min, and sample processing was performed immediately after arrival. Roe deer reproductive tracts with the ovaries and testes are shown in Figure 1.

### 2.1. Determination Methods

#### 2.1.1. Spermatozoa Characteristics

Epididymides were dissected and cleaned from connective tissue, and the samples were collected from the epididymides obtained post mortem. To diminish blood contamination, superficial blood vessels were cut previously, their contents were removed, and the cauda surface was thoroughly dried. Sperm were collected from both epididymides in each male in 1.5 mL tubes [16,22,23,24] by making several incisions in the cauda epididymis with a surgical blade and taking the white fluid emerging from the cut tubules. A preliminary assessment of the epididymal sperm involved an evaluation of the sperm motility and the sperm cell concentration in a Makler chamber. Semen samples with a total motility higher than 70% and a minimal concentration of 1 × 10^6^ spermatozoa/mL qualified for further analysis.

#### 2.1.2. Spermatozoa Concentration

The fresh sperm samples were diluted to a 1:4 ratio with phosphate-buffered saline (PBS) and incubated at 37 °C for 20 min. Then, 5 μL of each sample was placed in a pre-warmed Makler chamber (Sefi-Medical Instruments Ltd., Haifa, Israel) and examined from a minimum of five fields per sample. Additionally, for counting semen using the hemocytometric Burker method, the samples were further diluted in distilled water, which immobilized the sperm cells and facilitated counting.

#### 2.1.3. Sperm Motility Factors

Sperm motility factors were assessed using a computer-assisted sperm analysis (CASA) system (Hamilton-Thorne Research, HTR, IVOS version 12.3; Beverley, MA, USA), using the same settings as described in our previous study [22]. The CASA-determined sperm motility measure included the total motility (TMOT, %) and the progressive motility (PMOT, %) of fresh semen (FS) and frozen–thawed semen (TS).

#### 2.1.4. Sperm–Hyaluronan Binding Assay (HBA)

HBA is a diagnostic tool that uses dual hyaluronan-coated chambers for sperm sample analysis. It allows for distinguishing between mature spermatozoa that express hyaluronan receptors on their plasma membrane and immature ones that lack these receptors. The presence of receptors and the ability of spermatozoa to bind to hyaluronan is correlated with sperm maturity, increased chromatin integrity, decreased chances of aneuploidy, lower sperm DNA fragmentation, and better sperm DNA packaging. The proportion of mature spermatozoa with hyaluronan receptors was used to determine the maturation of the spermatozoa (BCT-HBA-10, CooperSurgical, Måløv, Denmark). FS and TS samples were loaded into an assay chamber and covered with a coverslip. Although TS maturation in vitro is performed by its incubation in the special medium, we wanted to compare the presence of hyaluronan receptors before incubation in the medium for its maturation in vitro. After 10 min of incubation at room temperature, the motility of spermatozoa was calculated in 10 grid squares per sample. The mean percentage of motile sperm per unbound motile sperm was determined via an HBA test in fresh semen to indicate sperm maturity.

#### 2.1.5. Sperm Morphology Assay

Smears were prepared from sperm samples diluted with PBS (1:4). The smears were air-dried and stained using the Giemsa staining method [25]. The morphological features of 200 sperm cells from each sample were evaluated under a phase-contrast microscope (Olympus CH45, Teltow, Germany). The results are presented as the percentages of sperm with a normal morphology in FS and TS and include the percentages of sperm with normal apical ridge acrosomes and the percentages of sperm with morphological defects of the head, midpiece, and tail.

#### 2.1.6. Sperm Viability

The viability of the sperm in semen suspensions was determined using flow cytometry in a Muse^®^ Cell Analyzer (Austin, TX, USA) using the Muse^®^ Count & Viability Kit, according to the manufacturer’s protocol, based on differential permeabilities of two DNA-binding dyes.

#### 2.1.7. Mitochondrial Membrane Potential

The mitochondrial membrane potential of the sperm in semen suspensions was determined using flow cytometry in a Muse^®^ Cell Analyzer using the Muse^®^ Mitopotential Kit according to the manufacturer’s protocol (Assay Quant Technologies Inc., Marlborough, MA, USA). This assay allows for the simultaneous measurement of two cell health parameters: change in mitochondrial potential, considered an early hallmark of apoptosis, and cellular plasma membrane permeabilization or cell death.

#### 2.1.8. Cryopreservation and Warming of Spermatozoa

Fresh sperm were diluted in a BioXcell extender (IMV, Steinheim, Germany) to a concentration of 1 × 10^6^ spermatozoa/mL and directly divided for analysis of the sperm motility: for a hyaluronan binding assay (HBA) and for cryopreservation. The white epididymal sperm fraction (without blood) from each male was used for cryopreservation; it was loaded into 0.25 mL straws and cooled gradually to 5 °C over 4 h, according to the protocol previously used for red deer [22]. The samples were frozen in static liquid nitrogen vapor 4 cm above the liquid nitrogen for 10 min using a Minicool 40PC (Air Liquide, Mascot, NSW, Australia). The freezing process was proceeded further by placing the straws 4 cm above liquid nitrogen as follows: +4 °C to −10 °C 5 °C/min, −10 °C to −100 °C/min, and −100 °C to −140 °C 20 °C/min. The straws were plunged into liquid nitrogen and stored for at least seven days prior to thawing and further analyses.

#### 2.1.9. Oocyte Isolation

Cumulus–oocyte complexes were collected by maceration of the ovaries of 16 does using scissors, separately for each female. The COCs were categorized as healthy, transitional, or atretic via microscope observations (Olympus SZX7, Teltow, Germany), based on the method developed by us earlier for red deer females [22]. Only healthy COCs were used for the study. Each doe contributed multiple COCs that were handled differently. The oocytes were divided; half of them were fertilized (*n* = 8) and the other half underwent viability measurements and vitrification without maturation (*n* = 8). The oocytes destined for IVF were placed in washing medium (IVF Bioscience, Falmouth, UK, 51002) and washed twice, and 10 oocyte drops were placed in 100 µL of IVM (IVF Bioscience, Falmouth, UK, 71001) with mineral oil (Sigma-Aldrich, Saint Louis, MO, USA, M8410) for 24 h.

#### 2.1.10. Oocyte Vitrification

Vitrification of the oocytes was conducted at the germinal vesicle stage according to the protocol described by Garg and Purohit [26]. Fresh oocytes (without undergoing in vitro maturation) were used for vitrification. The oocytes were washed in a washing medium and then placed in 50 µL of a mixture of freezing solution medium (FSM) and a basic solution medium (BSM) in a 1:1 proportion for 4 min. The freezing solution contained 15% ethylene glycol (Sigma-Aldrich, Saint Louis, MO, USA, E9129), 0.5M sucrose (Sigma-Aldrich, Saint Louis, MO, USA, S1888-500G), and 10% glycerol (Sigma-Aldrich, Saint Louis, MO, USA, G2025-100ML). The basic solution was made up of Dulbecco’s phosphate-buffered medium (DPBS; Sigma-Aldrich, Saint Louis, MO, USA, D8537-500ML) and 0.4% bovine serum albumin (BSA; Sigma-Aldrich, Saint Louis, MO, USA, A9418-50G). After 1 min, the oocytes were transferred to 1.5 mL Eppendorf tubes filled with the FSM and deposited into straws. Ten oocytes were placed in each straw (0.25 mL). The oocyte-filled straws were placed immediately in a manual freezing unit (Minitube, Tiefenbach, Germany, 15043/0636) and kept 5 cm above the liquid nitrogen for 2 min and then immersed in the liquid nitrogen and stored in a liquid nitrogen container. After ten days, the oocytes were thawed and assessed for vitrification efficiency using the MTT method.

#### 2.1.11. Evaluation of Vitrification Efficiency Using MTT

Before vitrification, one straw with ten oocytes from each individual was used for a viability test using the MTT colorimetric assay (Roche, 11465007001, Basel, Switzerland). This method measures cellular metabolic activity by assessing the reduction in MTT to purple formazan crystals, primarily by mitochondrial dehydrogenases, providing insights into the cytotoxicity levels in oocytes. Mitochondrial function is required for oocyte maturation, fertilization, and embryonic development, and measuring the activity of mitochondrial dehydrogenases is a useful method for evaluating all these processes. After vitrification, one straw of each individual was used to check the viability of the oocytes. The oocytes were warmed via incubation in IVM for 2 h at 38.5 °C in a 5% CO_2_ humidified air atmosphere. The oocytes from each straw were placed into 96-well culture plates. Then, 10 μL MTT reagent [3-(4, 5 dimethylthiazol-2-yl)-2, 5-di-phenyltetrazolium bromide] was added, and the oocytes were incubated at 37 °C for 4 h. As a positive control, 10 fresh oocyte samples were incubated with distilled water. Then, 100 μL of solubilization solution was added to dissolve the formazan crystals. Fresh oocytes were also added without distilled water as the control, to determine the effect of mitochondrial function with and without freezing. The absorbance wavelength of the formazan product measured in the MTT assay was 595 nm.

#### 2.1.12. In Vitro Fertilization

In vitro fertilization trials were conducted to assess the function of cryopreserved sperm and oocytes obtained post mortem. Prior to IVF, the straws of each male semen sample were prepared according to a previously described procedure [22]. After incubation, the semen was centrifuged at 200× *g* for 5 min, the supernatant was removed, and the sperm pellet was diluted in an appropriate volume of fertilization medium to a final concentration of 1 × 10^6^ motile sperm/mL.

Fresh mature oocytes were selected and directly fertilized, and immature oocytes were selected for maturation in IVM over 24 h. The matured oocytes were spherical in shape with a uniform zona pellucida and had a translucent cytoplasm and a polar body. The day IVF was performed was considered day 0. The embryos were separated from the cumulus cells by vortexing and were washed three times with washing medium. The conditions of the embryo culture were the same as in our previous study [22]. The numbers of isolated oocytes, COCs, cleaved embryos, expanded blastocysts, and embryos collected from day 6 to 9 of the culture were evaluated. The developmental stage and embryo quality were determined between day 6 and 9 of the culture based on the International Embryo Transfer Society manual. The quality of the blastocysts was scored as follows: grade A—excellent or good; grade B—fair or moderate; grade C—poor; and grade D—dead or degenerating.

### 2.2. Experimental Design

**Scheme 1:**
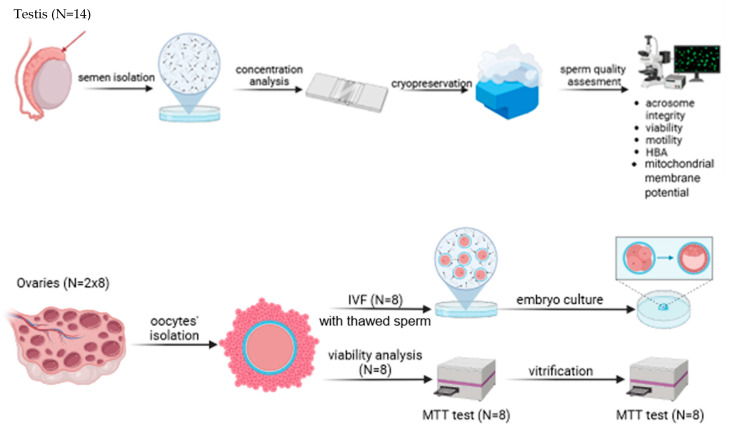
Schema of the experimental design.

The experimental design is shown on Scheme 1.

Each testis was weighed and the sperm volume was measured and the FS concentration was evaluated. The morphology, motility, viability, and mitochondrial membrane potential HBA and progressive movement were compared between FS and TS. We assessed the fertilizing ability of cryopreserved sperm via IVF.

The efficiency of the vitrification process was evaluated by calculating the viability of the oocytes (MTT). The results of MTT evaluation are presented as the average of the absorbance of all individuals before and after vitrification using SEM, as well as the percentage of viable oocytes calculated as the quotient of the average absorbance of all individuals before and after vitrification divided by the absorbance of the positive control multiplied by 100.

### 2.3. Statistical Analysis

Data were analyzed using Statistica version 13.1 software (Dell Inc. RoundRock, TX, USA) and expressed as the mean ± standard error. Analyses of the morphology, motility, and progressive movement of FS and TS were conducted using one-way ANOVA. Post hoc comparisons were performed using Tukey’s test. The results obtained for the oocytes before and after vitrification using MTT were compared using Student’s *t*-test. In all cases, the threshold of statistical significance was considered as *p* < 0.05.

## 3. Results

### 3.1. Experiment 1: Sperm Quality Analysis and Effectiveness of Cryopreservation

The weight of each testis ranged from 29 to 39 g (average: 32 ± 0.23 g) and 160 to 230 µL (average: 214 ± 26 µL) of sperm was obtained from one epididymis. The average sperm concentration of FS from the two testes of each individual was 4.95 × 10^9^/mL; the results of the HBA showed 61.8% in FS and 78.2% in TS (*p* < 0.05).

CASA showed a higher percentage of abnormal morphology measure (microscopic view of spermatozoa with oval head shape with well-defined acrosome, only one tail symmetrically attached with head) observed in FS compared to TS, contrary to progressive movement, which was better in TS compared to FS (*p* ≤ 0.001). Sperm motility was higher in FS compared to TS; however, the difference was not statistically significant (*p* > 0.05).

The viability of FS was 65.1 ± 4.1% and that of TS was 50.5 ± 3.4% (*p* < 0.05), and their mitochondrial membrane potentials were 54.3 ± 3.2% and 40.6 ± 4.1%, respectively (Table 1).

### 3.2. Experiment 2: Oocyte Quality and Effectiveness of Vitrification

**Figure 2:**
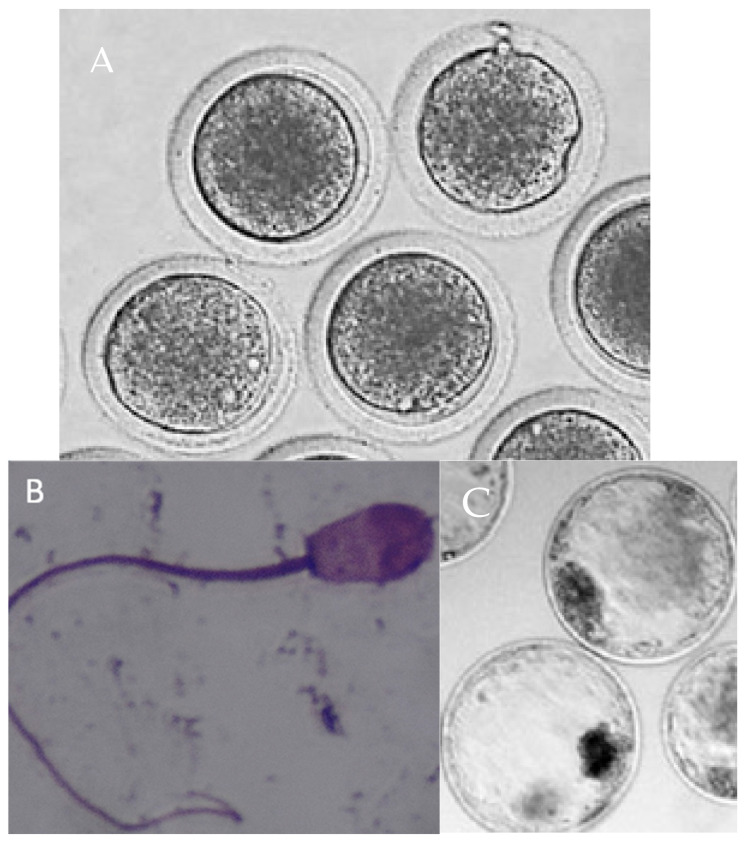
Representative images of roe deer oocytes (**A**), spermatozoa (**B**), and blastocysts (**C**). Magnification 100× (**A**,**C**) and 200× (**B**).

The average number of oocytes collected per individual was 38.9 (311 from 8 does in total). Only live oocytes were included in the experiments. The morphological appearance of the ooplasm was also assessed to select the oocytes (complete membrane, presence of lipid droplets). All oocytes were incubated for maturation for 24 h before IVF. A total of 150 viable COCs were used for IVF. Embryo cleavage was observed in 125 embryos, whereas the total number of expanded blastocysts was 24. The grades of blastocysts collected on days 6, 7, 8, and 9 were similar and thus, the results are presented as the average of the obtained blastocysts collected in the period between days 6 and 9 of the culture. The developmental rate of the blastocysts, calculated as the percentage of expanded blastocysts per cleaved embryo, was 19.2%. Regarding blastocyst quality scores, six blastocysts were graded A, four were graded B, six were graded C, and eight were graded D (Table 2, Figure 2—example pictures of oocytes, spermatozoa and blastocysts), in accordance the Association for the Study of Reproductive Biology (ASEBIR) criteria.

Two straws that each included 10 oocytes from one doe were evaluated for their viability using an MTT assay. After 10 days of vitrification, the oocytes were thawed. The calculated viability of the oocytes before and after vitrification revealed a high average IVF efficiency. For fresh oocytes, the viability was 98% (average absorbance of 0.553 ± 0.014), whereas after vitrification, it was 81% (average absorbance of 0.458 ± 0.042; *p* ≤ 0.001; Table 3).

## 4. Discussion

The use of reproductive biotechnology in wild ruminants is relatively rare. To the best of our knowledge, the first wildlife species in which reproductive biotechnology was applied is red deer (*Cervus elaphus*), which are, in some cases, bred as livestock, making the species more accessible for such technological interventions. Most ART was developed for domestic ruminants but can be modified and adapted to obtain the best qualitative results for wild ruminants as well, including for roe deer [11]. Nevertheless, until now, HBA (for fresh epididymal spermatozoa), mitochondrial potential tests (for TS), MTT (for oocytes), and IVF have never been conducted on roe deer. We successfully developed an ART for preserving the oocytes and spermatozoa of European roe deer and assessed its effectiveness. These studies could play a crucial role in enhancing the genetic diversity of the studied species. Moreover, the results may help in the preservation of gametes from other cervids, particularly species that are at risk of extinction.

Prieto-Pablos et al. [16] collected roe deer semen via electroejaculation and found the viability of the thawed spermatozoa to be between 54% and 67%, depending on the type of extender used. The viability of the spermatozoa of TS in our study was around 50%. However, we used a different extender, and the collected semen was epididymal, obtained post mortem. We conducted more in-depth analyses and also evaluated the motility and progressive movement of FS and TS. The improvement in these parameters in TS indicated the maturation of the spermatozoa, which is of relevant value [24]. We used BioXcell based on the high efficiency of this extender for IVF in red deer using TS [22]. Moreover, in our preliminary experiment, we tested the viability of roe deer FS using two different extenders without egg yolk: BioXcell (IMV, L’Aigle, France) and Andromed (Minitube, Tiefenbach, Germany). BioXcell was found to be the better extender, with a higher viability of FS after 2 h of incubation at 37 °C. Thus, we decided to use it for the main experiment. The quality of FS and TS was assessed by comparing their motility, progressive movement, and viability. The success rate of IVF was 19.2% with TS. The reason for the lower efficiency than that obtained for red deer in our previous study (22%) could be due to differences in the composition of the semen, which is species-specific, and the reproductive stage of the females used as oocyte donors for IVF [22]. Currently, antioxidants are used to improve the quality of thawed Cervidae semen [23,27,28]; however, such a procedure has never been used for roe deer. The viability of TS was 50.5%, with 53.7% showing motility and 35.6% showing progressive movement. The improvement in these parameters of TS compared to those of FS may be explained by the maturation of TS, especially the acrosome, which occurred after cryopreservation and the warming of the epididymal spermatozoa [24].

We used HBA to measure the fertility potential of the spermatozoa, in which hyaluronic acid surrounds the oocyte, only allowing spermatozoa with sufficient expression of specific receptors to fertilize it [29]. The percentage of HBA is lower in human epididymal spermatozoa than in ejaculates [30]. We showed that 61.8% of FS and 78.2% of TS potentially expressed receptors for fertilization. When applied to animal spermatozoa, such a test may be useful for insemination as an indicator of the integrity and maturity of the sperm membrane, especially for epididymal semen collected post mortem. However, further research into the maturation process of spermatozoa and the identification of hyaluronan receptors on spermatozoa would be valuable.

Our observations indicate that for the purpose of vitrification or IVF in roe deer, primiparous does (yearlings) or adults collected before the rut (e.g., in late spring or early summer from roadkill) are preferred, as more oocytes are obtained from such females compared to pregnant ones. Nevertheless, for the homogeneity of this study, the experimental oocytes included in this experiment were collected from pregnant adult does (3–5 years old; a single corpus luteum or two corpora lutea were present in the ovary) because we could not sample non-pregnant does or does during rut (mid-July–mid-August; restrictions due to the hunting calendar). Of all obtained oocytes, 81% were viable after vitrification. The rates of viable oocytes after vitrification were observed by different methods (microscopically, by evaluation of the nuclear status, apoptosis) to be 80.6% in cows [31], 8% in dogs [32], 66% in goats [33], and 42.4% in piglets [34]. In addition to measuring the viability of the oocytes, we evaluated the possibility of using vitrified oocytes for IVF (but we did not perform fertilization in the experiments in this manuscript). An additional concern linked to this procedure is the fact that we could only use TS, not FS (hunting season restriction). Semen was obtained from bucks that were harvested during the hunting season, earlier than does. The effectiveness of IVF with FS would likely be greater [35]. The developmental rate of the blastocysts was determined to be 19.2%, which was lower than in red deer (21.8%) [22]. In the preliminary experiment on roe deer and in a previous study on red deer hinds, we observed that the reproductive stage of a donor female influences the number of isolated oocytes [22]. We plan to expand our analyses of oocyte quality to monitor apoptosis and reactive oxygen species content and to determine the blastocyst quality. In this study, the results also show that the vitrification method was effective. Future studies should investigate how the maturation of oocytes before vitrification affects their factors after thawing. A study by Yodrug et al. [35] demonstrated that the vitrification of bovine oocytes at the meiotic stage significantly reduced blastocyst development after IVF, and vitrification at the GV stage resulted in improved blastocyst development compared to vitrification at the MII stage. All oocytes used in our study were at the GV stage, but in the next step of research, we will evaluate how the development of oocytes influences vitrification and IVF in roe deer. Further studies are also required to explore how the ecotype and reproductive stage influence the reproductive potential of roe deer.

## 5. Conclusions

Our study elucidates methods for isolating and freezing gametes, including oocyte vitrification and semen cryopreservation, from roe deer. We developed an ART that seeks to ensure a deer gene pool and stable deer populations, including those in decline, and to address the reproducibility crisis in wildlife animal science. We proposed a method for measuring the viability of oocytes and used them for IVF. Additionally, we determined the quality of FS and TS and showed the suitability of HBA for the quantification of spermatozoa in terms of maturation within the acrosome. One method of ex situ conservation is the establishment of cell and tissue banks. Cryopreservation of gametes is an extremely valuable tool in conservation programs for endangered species. The possibility of subjecting gametes collected after the death of an animal to the freezing process is particularly valuable, as this prevents the irreversible loss of genes of a given individual and allows the gene pool of an endangered population to be preserved. We intend to continue this line of research considering the condition and welfare of roe deer in different biotopes; nevertheless, our results prove that these methods used can be introduced into ART for the Cervidae family.

## Figures and Tables

**Table 1:** Quality factors of fresh and thawed epididymal spermatozoa of roe deer. Statistically significant differences are marked by different letters: a and b (*p* < 0.05).

		Fresh Semen	Thawed Semen
Concentration (×10^9^/mL)		4.95 ± 0.99	
HBA (%)		61.8 ± 4.8 ^a^	78.2 ± 5.3 ^b^
Morphology (%)		68.4 ± 0.6 ^a^	62.5 ± 2.1 ^b^
Motility (%)		49.2 ± 7.0 ^a^	53.7 ± 2.5 ^a^
Progressive movement (%)		19.4 ± 3.9 ^a^	35.9 ± 2.7 ^b^
Viability (%)		65.1 ± 4.2 ^a^	50.5 ± 3.4 ^b^
Mitochondrial membrane potential (%)	viable	54.3± 3.2 ^a^	40.6 ± 4.1 ^b^
	viable + dead	59.1 ± 2.2 ^a^	48.6 ± 3.2 ^b^

**Table 2:** Number of oocytes, COCs, and blastocysts collected post mortem in roe deer.

Doe Identification	Number of Oocytes	Number of COCs	Number of Embryos at Cleavage Stage	Number of Blastocysts (Quality Grades Number: A, B, C, D)				
				A	B	C	D	Total
D1	39	20	17	1	0	1	1	3
D2	38	20	16	1	1	0	1	3
D3	38	20	16	0	1	1	1	3
D4	40	20	17	1	0	1	1	3
D5	36	15	14	1	1	1	1	4
D6	45	20	16	1	1	1	1	4
D7	38	18	15	0	0	0	2	2
D8	37	17	14	1	0	1	0	2
***Average***	*38.9*	*18.8*	*15.6*	*0.75*	*0.5*	*0.75*	*1*	*3*
***Total number***	*311*	*150*	*125*	*6*	*4*	*6*	*8*	*24*

**Table 3:** Viability of roe deer oocytes assessed by MTT assay before and after vitrification. Values are presented as mean absorbance ± SEM and percentage of live oocytes. Different superscript letters (a, b) indicate statistically significant differences between groups (*p* ≤ 0.001).

Oocytes	Absorbance ± Sem	% of Live Oocytes
Before Vitrification	0.553 ± 0.014 ^a^	98
After Vitrification	0.458 ± 0.042 ^b^	81

## Data Availability

The datasets used and/or analyzed during the study are available from the corresponding author on reasonable request.
